# Unusual Instance of Primary Diffuse Large B-cell Lymphoma of the Colon

**DOI:** 10.7759/cureus.36083

**Published:** 2023-03-13

**Authors:** Amy Kiamos, Spencer G Streit, Abhinav Karan, Kimberly Boldig, Barrett O Attarha, Zoobia Kahn, Reeba Omman, Ron Schey, Bharatsinh Gharia

**Affiliations:** 1 Internal Medicine, University of Florida College of Medicine – Jacksonville, Jacksonville, USA; 2 Gastroenterology, University of Florida College of Medicine – Jacksonville, Jacksonville, USA; 3 Pathology, University of Florida College of Medicine – Jacksonville, Jacksonville, USA; 4 Gastroenterology, University of Florida Jacksonville, Jacksonville, USA; 5 Hematology Oncology, University of Florida College of Medicine – Jacksonville, Jacksonville, USA

**Keywords:** extranodal diffuse large b-cell lymphoma, extranodal lymphomas, primary colorectal cancer, colorectal cancer, non-hodgkin’s lymphomas, diffuse large b cell lymphoma (dlbcl)

## Abstract

Diffuse large B-cell lymphoma (DLBCL) commonly affects the gastrointestinal (GI) tract, although primary DLBCL rarely occurs in the colon. Primary colorectal lymphoma is a surprisingly rare diagnosis, accounting for a minute percentage of GI lymphomas and colorectal malignancies. We present an interesting case of an immunocompromised young adult female who was diagnosed with DLBCL confined to a cecum polyp after she underwent a colonoscopy for a GI bleed. The lymphoma presented endoscopically as a semi-sessile polyp in the cecum that was successfully removed. The patient was treated with appropriate therapy of rituximab, cyclophosphamide, doxorubicin, vincristine, and prednisone (R-CHOP).

## Introduction

Diffuse large B-cell lymphoma (DLBCL) is the most common type of non-Hodgkin lymphoma (NHL), accounting for approximately 35% of cases worldwide [1]. Patients with DLBCL typically present with a quickly enlarging mass evolving from one or more lymph nodes; although 40% of cases present with extranodal site involvement [2]. DLBCL may arise in any organ throughout the body while the most frequently affected extranodal location is the gastrointestinal (GI) tract [1]. While DLBCL has a preference for the GI tract, primary colorectal lymphoma remains a rare diagnosis comprising 3% of GI lymphomas and 0.1-0.5% of entire colorectal malignancies [3]. We present an interesting case of a young adult female who was diagnosed with DLBCL confined to a cecum polyp after developing a GI bleed.

## Case presentation

A 28-year-old female with a past medical history of systemic lupus erythematosus (SLE), lupus nephritis stage V, and aplastic anemia (9 years prior) presented to the emergency department complaining of nausea, vomiting, and diarrhea for three days. She described several episodes of non-bloody, nonbilious emesis per day and seven episodes of watery diarrhea per day. She was prescribed antibiotics (linezolid, ciprofloxacin, Flagyl) the week prior for an abscess. She denied fever, chills, night sweats, chest pain, abdominal pain, hematochezia, melena, or new rashes.

On admission, she was afebrile and tachycardic with a pulse of 123 beats per minute. Her physical examination was notable for mild abdominal tenderness to light palpation diffusely. Laboratory workup was significant for normocytic anemia with hemoglobin of 8.3 g/dL (baseline of 10 g/dL) and creatinine of 3.02 mg/dL (baseline of 1.7 mg/dL) (Table 1). She tested negative for Clostridium difficile and enterogenic pathogens (Salmonella, Shigella/enteroinvasive E.coli, Campylobacter, Shiga toxin) via nucleic acid amplification tests (NAAT).

**Table 1:** Laboratory Results at Initial Presentation

Lab Test	Result	Reference Range
White Blood Cell (WBC)	6.07 (thou/mm^3^)	3.4-10.8 (thou/mm^3^)
Hemoglobin (Hgb)	8.3 (g/dL)	11.1-15.9 (g/dL)
Mean Corpuscular Volume (MCV)	91.7 (fL)	79-97 (fL)
Platelets (plt)	338 (thou/mm^3^)	150-450 (thou/mm^3^)
Sodium (Na)	136 (mmol/L)	135-145 (mmol/L)
Postassium (K)	3.9 (mmol/L)	3.3-4.6 (mmol/L)
Chloride (Cl)	106 (mmol/L)	101-110 (mmol/L)
Carbon Dioxide (CO2)	20 (mmol/L)	21-29 (mmol/L)
Blood Urea Nitrogen (BUN)	16 (mg/dL)	6-22 (mg/dL)
Creatinine (Cr)	3.02 (mg/dL)	0.57-1 (mg/dL)
Glucose	91 (mg/dL)	71-99 (mg/dL)

**Figure 1:**
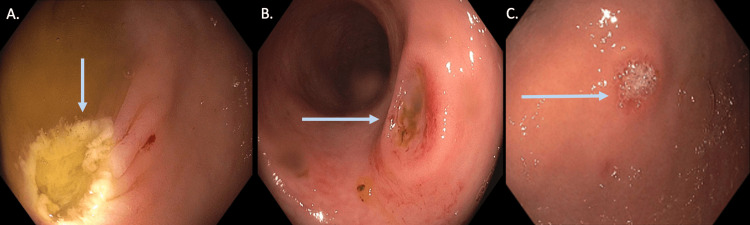
Endoscopic visualization of semi-sessile cecum polyp post polypectomy (A), sigmoid ulcer (B), and gastric ulcer (C)

On the second day of admission, she started having dark hematochezia accompanied by an acute drop in hemoglobin to 6.0 g/dL. Colonoscopy examination revealed a 5.0 millimeter (mm) semi-sessile polyp that was resected from the cecum, and two non-bleeding ulcers in the sigmoid and recto-sigmoid colon (5.0 mm, 6.0 mm, respectively) (Figure 1). Esophagogastroduodenoscopy (EGD) revealed two non-bleeding ulcers in the gastric antrum and fundus (5.0 mm and 4.0 mm, respectively) (Figure 1).

**Figure 2:**
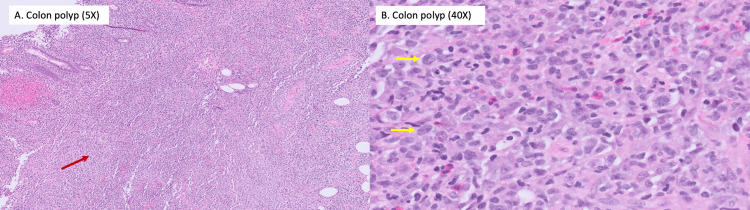
Hematoxylin and eosin (H&E) section of a cecal polyp, (A) Colonic mucosa (5x magnification) with a dense lymphoid infiltrate (red arrow), (B) Colonic mucosa (40x magnification) with a diffuse atypical lymphoid infiltrate The atypical lymphoid infiltrate is made of large cells with a high nuclear/chromatin ratio, irregular nuclear contour, vesicular chromatin, and occasional prominent nucleoli (yellow arrows).

Surgical pathology of the cecum polyp demonstrated an atypical lymphoid infiltrate primarily of large cells with a high nuclear-cytoplasmic ratio, irregular nuclear contour, vesicular chromatin, and prominent nucleoli (Figure 2).

**Figure 3:**
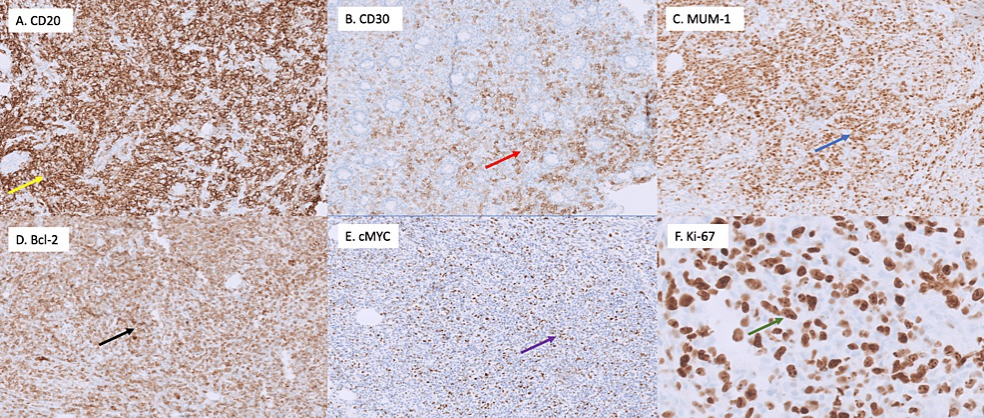
Immunohistochemical (IHC) stains of the atypical large cells were positive for (A) CD20 (yellow arrow), (B) CD30 (variable) (red arrow), (C) MUM-1 (blue arrow), (D) Bcl-2 (black arrow), and (E) c-MYC (40-50%) (purple arrow). (F) Ki-67 proliferation index was 80% (green arrow) cluster of differentiation 20 (CD20); cluster of differentiation 30 (CD30); B-cell lymphoma-2 oncogene (Bcl-2); multiple myeloma oncogene-1 (MUM-1); c-myelocytomatosis oncogene (c-MYC)

Immunohistochemical (IHC) stains of the atypical large cells were positive for CD20, CD30 (variable), B-cell lymphoma-2 oncogene (Bcl-2), multiple myeloma oncogene-1 (MUM-1), and c-myelocytomatosis oncogene (c-MYC) (40-50%) (Figure 3). IHC testing was negative for B-cell lymphoma 6 (Bcl-6), cyclin D1, anaplastic lymphoma kinase-1 (ALK-1), CD3, CD5, and CD10. Ki-67 proliferation index was 80% (Figure 3).

**Figure 4:**
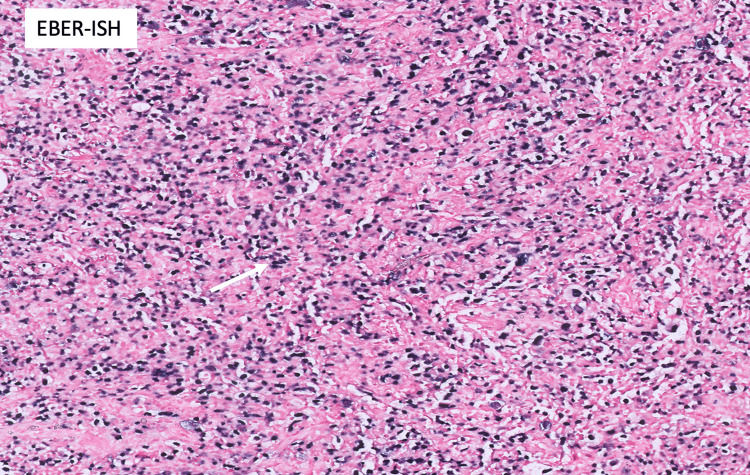
Epstein-Barr encoding region in situ hybridization (EBER-ISH) testing of atypical large cells was positive (white arrow)

Epstein-Barr encoding region in situ hybridization (EBER-ISH) was positive (Figure 4).

Overall, pathology findings were consistent with Epstein-Barr virus (EBV) positive DLBCL, activated B-cell type. The stomach ulcers were consistent with active gastritis with a negative Helicobacter (H.) pylori stain while the colon ulcers showed focal active colitis with negative cytomegalovirus testing.

Oncology was consulted and she underwent a staging evaluation. Computerized tomography (CT) of the neck, chest, abdomen, and pelvis demonstrated no pathologically enlarged lymph nodes. Bone marrow biopsy demonstrated hypocellular marrow (40% cellularity) with no evidence of acute leukemia, lymphoma, or myelodysplasia, which was confirmed with flow cytometry. She was transfused with irradiated blood with an improvement of her acute blood loss anemia. She was diagnosed with stage IV diffuse large B-cell lymphoma with an international prognostic index (IPI) of 2 points. She was started on chemotherapy for curative intent with rituximab 375 mg/m^2^, cyclophosphamide 750 mg/m^2^, doxorubicin 50 mg/m^2^, vincristine 2 mg, and prednisone 125 mg (R-CHOP) for a total of six cycles.

Unfortunately, after receiving a few cycles of chemotherapy, the patient developed multiple complications requiring hospital admissions. Her clinical course has been complicated by pancytopenia, symptomatic anemia, neutropenic fever, sepsis from a chemotherapy port infection, and chemotherapy-induced peripheral neuropathy. She has not completed the six total chemotherapy cycles due to repeat hospital admissions and continued neutropenic infections.

## Discussion

The GI tract is the most common extranodal site for secondary involvement of NHL; however, primary NHL of the GI tract only accounts for 10-15% of NHL [4]. Primary GI NHL frequently arises in the stomach (60% of cases) followed by the small intestine [5]. Interestingly, primary colorectal lymphoma is a rare diagnosis. Primary colorectal lymphoma is infrequently reported accounting for 1.4% of NHL and 0.1-0.5% of all colorectal cancers [3-4]. The cecum is the most common site (60-74% of cases) due to the presence of excess lymphoid tissue [3].

DLBCL is an aggressive type of NHL that most commonly affects males with a mean age diagnosis of 55 years old [6]. DLBCL is the most common histological subtype of primary colorectal lymphoma [4]. Risk factors associated with DLBCL include inherited immunologic deficiency diseases (e.g., common variable immunodeficiency, ataxia-telangiectasia), autoimmune diseases (e.g., SLE, Sjogren’s disease, rheumatoid arthritis), immunosuppression by drugs (e.g., organ transplant patients), and viruses (e.g., human immunodeficiency virus (HIV), hepatitis C (HCV), human herpes virus 8 (HHV8), EBV) [2]. Histologically, it is characterized by large lymphoid cells with a high nuclear-cytoplasmic ratio, irregularly shaped nuclei with prominent nucleoli, and basophilic cytoplasm [6]. Immunophenotypic features of the neoplastic B-cells express markers such as CD19, CD20, CD22, and CD79a [1]. Immunohistochemical profiling can help depict prognostic factors: expression of MUM-1, myelocytomatosis oncogene (MYC), Bcl-2, and B-cell lymphoma oncogene-6 (Bcl-6) tend to have unfavorable outcomes, whereas the expression of CD10 and cellular FLICE-inhibitory protein (cFLIP) tend to have better overall survival [1,7]. Our patient’s histology was positive for CD20, CD30 (variable), Bcl-2, MUM-1, and c-MYC (40-50%). The overexpression of Bcl-2 and MUM-1 proteins in our case supported a double expressor lymphoma, which indicated a poor prognosis and an aggressive clinical course.

Clinically, colorectal lymphoma can present with symptoms such as abdominal pain, weight loss, abdominal mass, bowel obstruction, or lower GI bleeding [8-9]. Immunosuppression is a known risk factor for the development of primary colorectal lymphomas although the mechanism is not well understood. Patients with primary and secondary forms of immunodeficiency (e.g., HIV, long-term corticosteroid use, post-transplantation, and immunosuppression following chemotherapy) have been associated with an increased risk of developing GI lymphomas [10-11]. The most common extranodal site of HIV-associated NHL is the GI tract occurring in 30-50% of cases; however, with the introduction of highly active antiretroviral therapy (HAART), there has been a significant decrease in cases reported [10]. There have been some studies reporting irritable bowel disease as a predisposing condition to GI NHL, although the association has been somewhat controversial [3,10]. Patients with SLE have an increased risk of developing cancer, particularly lymphomas, likely in the setting of immune system dysregulation and chronic inflammation [12]. In patients with SLE, the most common lymphoma reported is DLBCL accounting for 37-62% of cases [12]. Our patient was taking immunosuppressive agents with hydroxychloroquine, mycophenolate mofetil, and prednisone for her SLE and lupus nephritis stage V putting her at an increased risk for developing primary colorectal lymphoma.

Treatment options available for colorectal DLBCL include surgical intervention, chemotherapy, and/or radiation. While surgical resection is essential for the role of diagnosis, the role of surgical intervention for treatment is debated. Some studies have shown improved outcomes with surgical resection followed by chemotherapy, although some authors believe surgery should only be indicated for emergency situations like hemorrhage, obstruction, or perforation [13-15]. Surgical resection followed by chemotherapy has been shown to have improved outcomes in patients with a localized disease more amendable to resection [15]. An individualized surgical treatment plan is recommended to optimize the highest chance of cure. The standardized treatment is immunochemotherapy with the regimen of rituximab, cyclophosphamide, doxorubicin, vincristine, and prednisone (R-CHOP) [16-17]. Studies have shown that the addition of rituximab to the gold standard regimen of cyclophosphamide, doxorubicin, vincristine, and prednisone (CHOP) therapy has improved outcomes and survival rates in patients with DLBCL [18-19]. Radiation is considered based on response to systemic chemotherapy or the degree of tumor burden [17]. The prognosis for DLBCL can vary depending on the patient and the burden of the disease. Prognostic factors include the patient’s age, performance status, tumor burden, stage of disease, site of extranodal involvement, and specific laboratory values (lactate dehydrogenase, B2-microglobulin) [2]. The cure rate is higher in patients with a limited disease while patients with advanced or symptomatic disease have approximately 50% progression-free survival [2].

## Conclusions

Primary colorectal lymphoma is a rare diagnosis. This case highlights a rare presentation of primary colorectal DLBCL in an immunocompromised patient. The patient was diagnosed with DLBCL after she underwent a colonoscopy for a GI bleed with resection of a cecum polyp. Immunosuppression and SLE have been shown to increase patients' risk of developing GI lymphomas. It is important to keep lymphoma in the differential for these at-risk patients.
